# Annotations

**Published:** 1892-10-08

**Authors:** 


### ANNOTATIONS.

**Cholera: One Step Further.**

Professor Hankin, as our readers will remember, voluntarily submitted himself a few weeks ago to "cholera inoculation" at M. Pasteur's Paris Institute. All lovers of medical progress gave him their sympathy and respect on that occasion. Since then, by way of testing the value of the inoculation, he has advanced a step further in self-devotion to a great cause. The "Virus exalté" of M. Pasteur he has had prepared for drinking, and has bravely swallowed a portion. The "Virus exalté," our readers will remember, is a cholera virus artificially intensified till it is said to be twenty times stronger than the normal. Guinea-pigs inoculated with it speedily died. The effect produced upon Mr. Hankin by this potion was no more than a slight diarrhœa. The presumption, therefore, is that Mr. Hankin is at least to some extent "protected" by the inoculations he has undergone. The protection, however, is manifestly not complete, for if it had been there should have been no diarrhœa at all. The inoculation itself was so very recently performed that we may reasonably suppose Mr. Hankin to have been at the stage of "maximum immunity." Yet in spite of this a "slight diarrhœa" has resulted from the swallowing of the virus. There is another point, too, to be considered, and that is the quantity of the virus actually swallowed. We are not informed on this point, though we may believe that it was very small, because no sane person would be justified in swallowing a large quantity of a virulent poison by way of a first experiment. For what Mr. Hankin has already done science and medicine are profoundly grateful. They cannot in conscience urge him to do more; but, as we pointed out in a former article, the experiment of experiments still remains to be carried out. That experiment would be that a large number of Pasteur's inoculated subjects should go to a district infected with epidemic cholera, and should there be inoculated with the natural virus, should drink the natural virus, and should otherwise experiment with the natural virus in every way that science could suggest. We do not ask any scientific enthusiast to do this; but we say and repeat that it is the only kind of experiment which would be of a really conclusive nature.

**Aid to the Injured.**

A book on an important subject, of which the price is only twopence, ought to be looked at with almost suspicious criticism. One naturally asks, Can it be really worth anything at such price? The National Health Society has just published the fifth edition of Dr. Willingham Gell's "Aids to the Injured and Sick," at the price of twopence. A careful study of the little work establishes one conclusion in the present critic's mind—viz., that it is the best twopennyworth of literature he ever remembers to have seen. In the first place Dr. Gell has compressed into six quarto pages of easily-readable print an intelligible description of the leading features of the anatomy and physiology of the human body. This is really a triumph of the condenser's art. So well is the description done, that any intelligent person, who may take the pains to master, it will have quite a serviceable comprehension of the structure and functions of the human organism. After the anatomy there comes the detailed instruction for dealing with injured and sick persons until the attendance of a doctor can be secured. This instruction is of such a kind that it appeals at once to the trained mind of the doctor; but it is at the same time so simple, and so entirely in sympathy with the untrained mind of the layman, that it can be comprehended and put into practice by the most ignorant person who is endowed with a little common sense. Here, for example, are two or three points which show a doctor that the little book is no mere compilation, but the work of a practical and sympathetic workman: "Do not *pull* anything off an injured part, but *cut* or *rip up* the seams of trousers, coat, &c.; use as sharp a knife or scissors as you can get." Have we not all seen thoughtless or too economical relatives pulling away with might and main at trousers or boots, inside which was a crushed foot or a torn and smashed leg? Here is another sample of the extreme practicality of this little twopenny manual: "In undressing an injured person to put him to bed, take the clothes off the sound side first; then remove them gently from the injured side, cutting the seams, &c." Stimulants, as everybody knows, are almost invariably needed on the first approach of consciousness after an accident; and almost everybody knows that tea, coffee, broth, wine, whisky, brandy, and sal volatile are suitable stimulants. But there are two practical points about the administration of stimulants which most lay persons, and even some doctors and nurses seem to forget: the one is that they should be given slowly; and the other that they should be given hot, though weak. These are points which the author puts most clearly, and insists upon. They are samples of the whole book. It is in no spirit of puffery that we say that such a little work ought to be in every home.

**The "Pall Mall Gazette" Inside the Hospitals.**

A couple of articles, clever, amusing, but also pathetic, have recently appeared in the *Pall Mall Gazette* which reveal a good deal of the inner life of one of our general hospitals. The writer writes with knowledge, and one cannot but think with personal experience. The children he describes appeal to our tenderest sympathies; the mothers who have reared them, and who generally accompany them to the hospital, appeal to quite a different set of feelings. Ignorant, inconsequent, and impudent, they make one long for some power of stern discipline which would curb their intolerable tongues. One cannot help wondering what sort of wives these mothers can be; what sort of homes they make unbearable with their presence. Still more is one disposed to ask how children brought up under such auspices can ever be anything but ignorant, mindless, and worthless creatures as long as they live. But perhaps there is another side to the question. We must recollect that the physician in the out-patient department has a thankless time of it, and may feel a little bitterly. He gives instructions which he quite despairs of ever seeing carried out; he sees whole worlds of good scientific service rendered useless and nugatory by the perverse wilfulness and stupidity of patients, and those who are in charge of patients. He gives many hours of his valuable time every week to creatures who are not only ungrateful, but who will not even take him at his word and use the science he so liberally places at their disposal. It is said that the hospital physician has his *quid pro quo*. No doubt he has an object in view; but can any hope of future reward, however great, compensate him for the hours of wearing and thankless toil, and the disgusting filthiness, and still more disgusting rudeness, he is so constantly called upon to endure? The effect of the *Pall Mall's* articles is to paint on the tablets of the mind a patient man of science trying to do the noblest of work under conditions of absolute impossibility. One feels that if out-patient work is really such as is described, then the whole out-patient department, as at present conducted, ought to be swept away in a Noachian deluge of public indignation. The *Pall Mall* has rendered excellent service in thus calling public attention to one of the most unwholesome and indefensible blots on our whole hospital system.

